# Pancreatico-pericardial fistula as a complication of chronic pancreatitis

**DOI:** 10.12688/f1000research.3-31.v1

**Published:** 2014-01-29

**Authors:** Camille Anne Sommer, C. Mel Wilcox

**Affiliations:** 1 Division of Gastroenterology and Hepatology, University of Alabama at Birmingham, Birmingham, AL, BDB 380, USA

## Abstract

Pancreatico-pericardial fistula is an extremely rare complication of chronic pancreatitis. We present a case of a 58-year-old man who presented with syncope. Transthoracic echocardiogram revealed a pericardial effusion with tamponade physiology. Pericardiocentesis and pericardial fluid analysis demonstrated a lipase level of 2321 U/L. Subsequently, an endoscopic retrograde cholangiopancreatography (ERCP) was performed, confirming the presence of a pancreatico-pericardial fistula (PPF) from the distal body of the pancreas. A pancreatic duct stent was placed across the duct disruption on two separate occasions; however, despite stent placement, the patient continued to re-accumulate pericardial fluid and deteriorated. While rare, PPFs may complicate chronic pancreatitis, may not respond to pancreatic duct stenting and may portend a poor prognosis.

## Introduction

Pancreatic fistulas are a well-recognized complication of pancreatitis. They result from leakage of pancreatic enzymes from a disrupted pancreatic duct associated with acute or chronic pancreatitis, partial pancreatectomy, or injury to the pancreatic duct during surgery. Other causes include pancreatic biopsy and blunt abdominal trauma. Fluid collections develop from persistent leakage of pancreatic enzymes with subsequent erosion through the wall of the bowel and into a nearby hollow viscus (colon, duodenum, stomach, or esophagus), where they communicate with peritoneal or pleural cavities causing effusions in the pleural space, mediastinum, lesser sac, retroperitoneum, or perihepatic space. We report a rare case of pancreatico-pericardial fistula (PPF) presenting as syncope in a patient with chronic alcoholic pancreatitis.

## Case report

A 58 year-old black male presented with syncope related to orthostatic hypotension. He had sustained a laceration and multiple facial fractures involving the zygomatic arch and right orbit. He reported feeling weak with dyspnea on minimal exertion for several days prior to this syncopal event. He denied any abdominal pain, nausea, vomiting or chest pain. He had a history of delirium tremens post alcohol cessation and previous episodes of acute pancreatitis over the last two years. Prior surgical history was notable for an exploratory laparotomy in 1985 after sustaining a gunshot wound to the abdomen. He was on no medications on admission. He had an ongoing history of alcohol dependence (3 glasses of whisky daily) and was drinking earlier on the day of his presentation (ethanol level 178 mg/dl). He denied any family history of pancreatic disease.

On admission, his vital signs were normal without tachycardia or hypotension. Facial trauma was evident. On auscultation he had rales at both lung bases. There were no abnormalities noted on his cardiovascular exam. He had moderate ascites with an obvious fluid wave but no hepatosplenomegaly. Pertinent laboratory findings included: hemoglobin 7.7 g/dL (normal range 14–18 g/dl) and hematocrit 23.5% (normal range 40–54%). The remainder of his blood count was normal. His total bilirubin was 1.3 mg/dl (normal range 0.3–1.2 mg/dl), aspartate aminotransferase (AST) 130 U/L (normal range 15–46 U/L) and alanine aminotransferase (ALT) was 28 U/L (normal range 11–66 U/L) with albumin of 2.2 g/dl (normal range 3.5–5 g/dl). Alkaline phosphatase (ALP) was within normal limits. Electrolytes on admission were also normal. Prothrombin time (PT) and INR were 22.9 seconds (normal <12.5s) and 2.08 (normal <1.11), respectively. His serum amylase concentration the day after admission was 386 U/L (normal range 30–110 U/L). He was admitted to the Medicine Intensive Care Unit for treatment of syncope, pancreatitis, and anemia.

Computed tomography (CT) of his head revealed multiple fractures involving the right posterior lateral wall of the right maxillary sinus, zygomatic arch and lateral wall of the right orbit, without intracranial hemorrhage. The admission chest X-ray demonstrated cardiomegaly without evidence of pneumonia or pulmonary edema. Overnight, the patient became confused and tachypneic and his oxygen saturation fell to 85%. A transthoracic echocardiogram was subsequently performed on the day after admission to evaluate for dilated cardiomyopathy or pericardial effusion. A large pericardial effusion was seen with systolic right atrial collapse, diastolic right ventricular collapse and left atrial collapse. Pericardiocentesis was performed with removal of 4.2 liters of straw-colored fluid and resolution of the tamponade. Fluid analysis showed: red blood cell count 573/mm ^3^, white blood cell count 13/mm ^3^, amylase 633 μ/L, and lipase 2321 μ/L. Despite pericardiocentesis, a repeat chest X-ray on the day after admission showed worsening cardiomegaly and a repeat echocardiogram showed re-accumulation of the fluid and an additional 1800 cc was drained over the next two days; a pericardial drain was subsequently placed.

**Figure 1.**
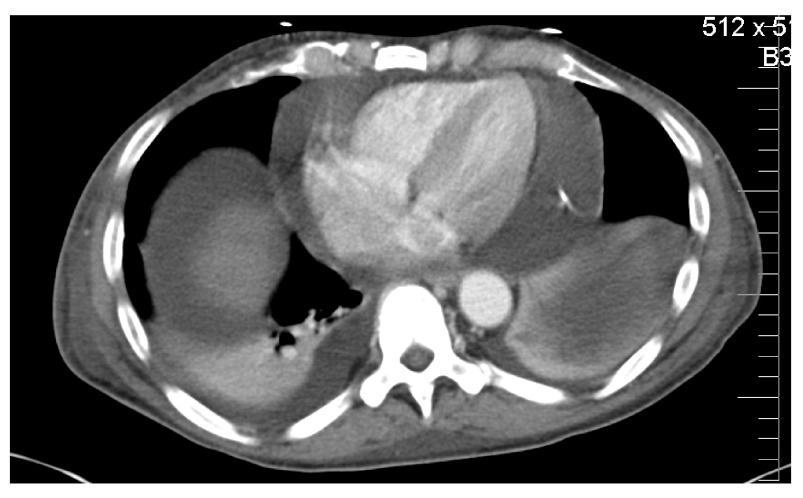
CT Chest. Large pericardial and pleural effusions. The tip of the drain is shown in the pericardial space.

**Figure 2.**
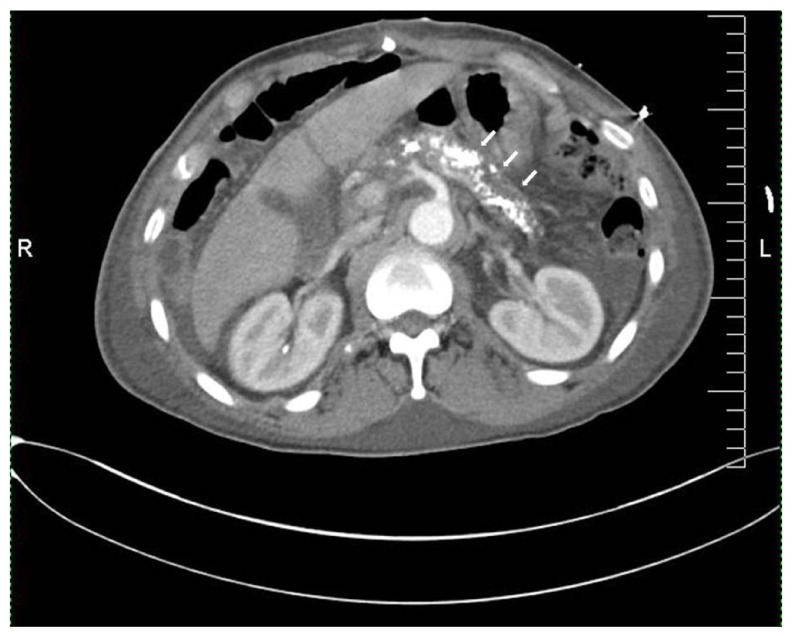
CT Abdomen. Calcifications within the pancreas are shown by arrows.

An abdominal ultrasound three days after admission showed mild ascites and a cirrhotic liver without portal vein thrombosis. The patient was also found to have hepatitis C by blood testing. A CT of the chest, abdomen and pelvis revealed a persistent large low-attenuating pericardial effusion with the pericardial drain entering the left paramedian subcostal region with the tip terminating at the level of the ascending aorta ( Figure 1); large bilateral, left greater than right, pleural effusions were also present. Changes of chronic pancreatitis were present with irregular and dilated ducts with numerous calcifications ( Figure 2). There was no evidence radiographically of acute pancreatitis. The pancreatic parenchyma was homogenously enhancing. There was a moderate amount of low-attenuating free intraperitoneal fluid but no discrete peripancreatic fluid collection or stranding.

The patient continued to have a re-accumulating pericardial effusion with over 12 L removed over the ensuing several days. The cardiothoracic surgery service was consulted regarding a pericardial window, but the patient was felt to be a poor surgical candidate and too high-risk for the procedure given his co-morbidities. A technetium-labeled macro-aggregated albumin shunt study was performed five days after admission through the pericardial drain to evaluate for communication and there was no scintigraphic evidence for radiotracer communication from the pericardium into the abdomen or pelvis.

**Figure 3.**
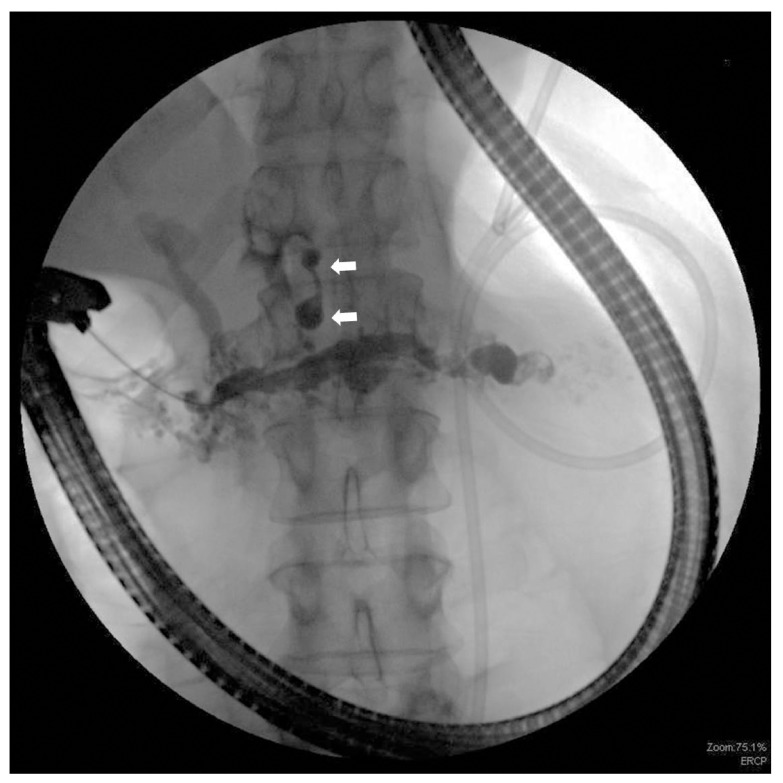
Calcifications consistent with chronic pancreatitis. Pancreatic fistula is shown by the arrows.

**Figure 4.**
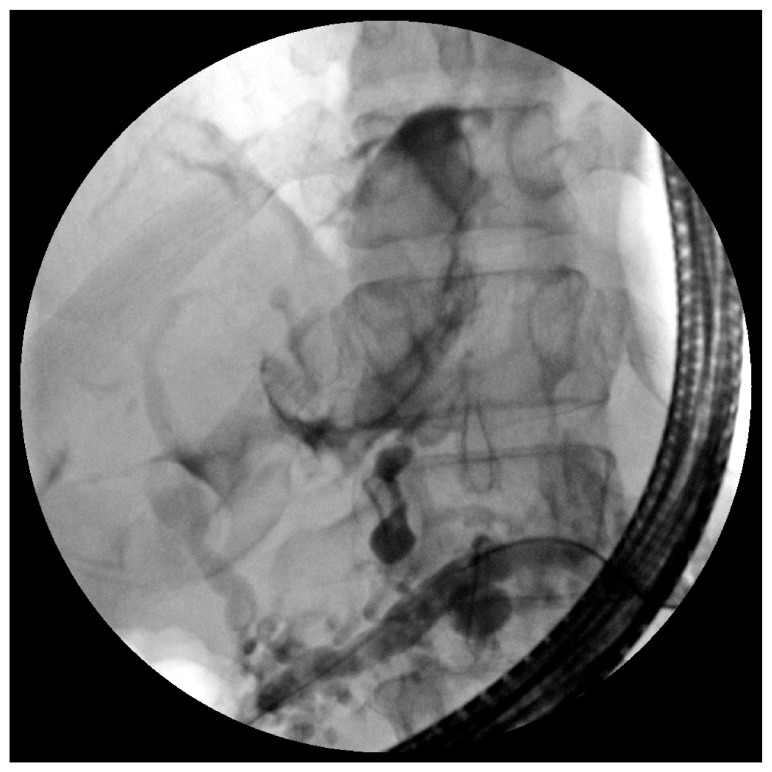
Pancreaticopericardial fistula coursing cranially surrounded a circular sac, consistent with the pericardium.

Despite a pericardioplasty, the patient continued to have persistent pericardial drainage. Endoscopic retrograde cholangiopancreatogram (ERCP) was performed for suspected PPF. A scout image showed numerous calcifications throughout the pancreas that were consistent with chronic pancreatitis. After biliary sphincterotomy, the pancreatic duct was selectively cannulated and revealed numerous small stones and a diffusely dilated pancreatic duct to the tail. A leak was observed in the distal pancreatic body just proximal to the pancreatic neck ( Figure 3). The fistula was seen coursing cranially surrounding a circular structure, consistent with the pericardium ( Figure 4). As the ERCP contrast agent was continually injected under fluoroscopy, cardiac contractions were observed. A 7-French × 15 cm pancreatic duct stent was placed to the tail, bridging the leak.

Nevertheless, the patient continued to have 3 L of output from the pericardial drain daily. A repeat CT did not show any focal peripancreatic fluid collection or tract and no visible collection or tract was seen between the pancreas and diaphragm for percutaneous drain placement to be possible. A second ERCP was subsequently performed with exchange of the stent. The fistulous tract was again noted from the neck, tracking cranially, and a 7 Fr pancreatic stent was again placed bridging the leak.

The patient continued to decline despite maximal medical therapy. While hospitalized, over 50 L of fluid was removed from the pericardial drain. He did not respond to pericardial drainage nor to treatment of pansensitive *Escherichia coli* that was cultured from the pericardial fluid. He was also treated with antibiotics for concurrent pneumonia and a urinary tract infection. Eventually, the patient opted for a change in his goals of care to comfort care and was transferred to hospice and expired.

## Discussion

Disruption of the pancreatic duct leads to leakage of pancreatic juice and formation of an acute fluid collection or pseudocyst. Communication to other structures can occur either indirectly, originating from a pseudocyst, or directly in the form of fistulas. Internal pancreatic fistulas result from erosion of pancreatic fluid into adjacent or distant organs whereas external fistulas drain into a defect in the skin. If the communication occurs anteriorly into the peritoneal cavity, pancreatic ascites may occur.

In most reported cases of internal pancreatic fistulas, thoracopancreatic communication occurs into the pleural space causing a pleural effusion and patients usually present with dyspnea ^1^. Although there have been reports of mediastinal extension of pseudocysts ^2– 5^, to our knowledge only four cases of PPFs have been reported in the medical literature ^6– 9^.

The presenting symptoms can be variable depending on the location and size of the communication. Thus, patients may present with dyspnea, chest pain, palpitations, or dysphagia; sometimes with hemoptysis, acute respiratory compromise, or cardiogenic shock. Our patient presented with syncope and reported no chest or abdominal symptoms. It was only when his pericardial effusion was analysed and found to have elevated amylase and lipase concentrations that a pancreatic origin was suspected.

A number of techniques may be used to establish the diagnosis of pancreatic fistula. Cross-sectional abdominal imaging by computed tomography may often be suggestive, especially if a pancreatic pseudocyst or peripancreatic fluid is observed. More recently, magnetic resonance cholangiopancreatography has been used to delineate the pancreatic duct and even to identify the fistulous tract. A variety of radiologic imaging studies were performed on our patient, including abdominal ultrasound, transthoracic echocardiogram, CT, and a technetium-labeled macroaggregated albumin shunt study, none of which were able to detect or confirm the presence of the pancreatico-pericardial fistula. ERCP is the diagnostic test of choice and offers the potential for therapy for fistulas as stenting the disrupted duct is a viable and effective therapeutic option ^10– 15^.

The treatment of pancreatic fistulas is based primarily on case reports and small observational studies ^16^. Optimizing nutritional status and limiting oral intake may be effective with small leaks. Octreotide, a potent inhibitor of pancreatic secretion, has also been used in some small studies with efficacy ^16^. The therapy most commonly recommended, however, is endoscopic stenting by ERCP ^17, 18^. One randomized study in patients with pancreatic duct disruption associated with necrotizing pancreatitis, however, found no significant difference between conservative and endoscopic treatment ^17^. In the study by Kozarek *et al.* ^19^, of 18 patients with pancreatic duct leaks (14 of which had associated fluid collections) who were treated with transpapillary pancreatic duct drains or stents, 7 patients required surgery for ongoing pancreatic pain or residual/recurrent fluid collections. The complications were the result of exacerbated symptoms of pancreatitis and stent occlusion leading to recurrent pancreatitis or recurrent duct blowout with pseudocyst. Covered self-expanding metal stents have been successfully used in the main pancreatic duct to manage duct disruptions and leaks ^20^. A retrospective study of those with pancreaticopleural fistula who underwent ERCP and stenting as the primary treatment found successful treatment in only one of eight cases, mostly due to abnormal or difficult anatomy ^21^. Location of duct disruption has been the only suggested predictor of who would best benefit from stenting for pancreatic duct leak ^1^. Despite identifying the ductal leak and stenting twice, the leak in our patient did not resolve. If the leak persists after 2–4 weeks of conservative and endoscopic therapy, surgery may be required depending on the site of the leak. In one patient, surgical detachment of the fistula and Roux-en-Y decompression of the pancreatic duct resulted in a cure of the condition ^6^.

In the medical literature, there is a report that described the case of a 16 year-old male who was found to have tropical calcific pancreatitis and underwent a lateral pancreatico-jejunostomy with Roux-en-Y loop performed after initial pericardiocentesis and was reported to be doing well six months later ^7^. Another report described an adult patient who underwent an elective Roux-en-Y pancreatico-jejunostomy without complication and remained symptom-free two years after surgery ^8^. Unfortunately, our patient had untreated cirrhosis due to hepatitis C and alcohol misuse, and had continued to consume alcohol up until the day of admission, and was deemed a poor surgical candidate. Another more recent case describes a PPF diagnosed by ERCP in a patient with acute alcoholic pancreatitis with successful stent placement in a disrupted pancreatic duct. Prior to diagnosis, the patient had been treated with intrapericardial triamcinolone and octreotide, but the pericardial effusion reccurred ^9^.

Pancreatico-pericardial fistula is a very rare complication of pancreatitis and rapid and accurate diagnosis of this life-threatening complication is crucial. As our case demonstrates, endoscopic therapy may not be effective.

## Consent

As the patient died and it was not possible to locate the patient’s family, the authors obtained written permission for publication of clinical details and clinical images from Prof. Klaus Monkemuller, Chief of Endoscopy at the University of Alabama at Birmingham Hospital, where the patient was managed. Prof. Monkemuller has confirmed the absence of patient identifiers ensuring protection of any patient related information.
