# Supplementary material for: Pancreatico-pericardial fistula as a complication of chronic pancreatitis
Source: F1000Res. 2014 Jan 29;3:31. [Version 1] doi: 10.12688/f1000research.3-31.v1 (PMC3945763; doi:10.12688/f1000research.3-31.v1)
Supplement: Peer review history

# Peer review: Referee response for version 1

**Published:** 2014-02-03

**Authors:** Matthew DiMagno, Erik-Jan Wamsteker

**Affiliations:** 1 Division of Gastroenterology, University of Michigan, Ann Arbor, MI, USA

This is a well written case report about a patient presenting with syncope due to a pancreatic-pericardial fistula, a rare complication of chronic pancreatitis that has been reported for only a handful of patients in the literature. Multiple noninvasive diagnostic tests failed to detect the fistula. ERCP proved diagnostic, showing a leak of contrast from the distal pancreatic body coursing to and surrounding a circular structure in the chest. There are no controlled data or guidelines for management of pancreatic-pleural fistula but general treatment options include watchful waiting for spontaneous resolution, medical therapy (octreotide, nasojejunal feeding, total parenteral nutrition), or drainage internally or externally by endoscopic, percutaneous, or surgical approaches. The patient was too symptomatic from the pericardial effusion to offer observation alone and he was not fit for surgery due to his comorbid illnesses. Endoscopic stent placement is a very reasonable option and potentially a first-line therapy based on the excellent outcomes reported by case series for management of pancreatic-pleural fistula and in the single of 4 cases of pancreatic-pericardial fistula offered this treatment modality. It should be noted that those having a fistula between the pericardium and a (peri)pancreatic pseudocyst, which was absent in this case, may be offered endoscopic cystenterostomy. Unfortunately, this patient failed to respond to endoscopic stenting across the body of the pancreatic duct, based on having an unremitting, large pericardial drain output of approximately 3 Liters per day of pancreatic juice. Why this particular patient did not respond is unclear. It should be emphasized that the patient underwent stent change, essentially excluding a clogged or malpositioned stent as a cause for failed therapy. Moreover, we speculate that fistula pathophysiology and perhaps the response to transpapillary or bridging pancreatic duct stenting differs between pancreatic-pleural and pancreatic-pericardial types of fistula and that the extreme, high output fistula in this particular patient would doom any treatment modality that did not include surgery.

We have read this submission. We believe that we have an appropriate level of expertise to confirm that it is of an acceptable scientific standard.

# Peer review: Referee response for version 1

**Published:** 2014-02-03

**Authors:** Kevin McGrath

**Affiliations:** 1 Division of Gastroenterology, Hepatology, and Nutrition, University of Pittsburgh Medical Center, Pittsburgh, PA, USA

This case report serves to remind us of a rare but potentially devastating complication of chronic pancreatitis, and one that unfortunately does not respond well to endoscopic management.  Given the comorbities, this patient was not appropriate for surgical management. 

The authors also discuss the role of octreotide in the management of pancreatic fistulae. It would be interesting to know whether octreotide was used in conjunction with pancreatic duct stenting in this case. Given the drainage volume, I suspect it would have been of little benefit if used.

I have read this submission. I believe that I have an appropriate level of expertise to confirm that it is of an acceptable scientific standard.
